# Sudden unexpected death in epilepsy: A bibliometric overview

**DOI:** 10.3389/fneur.2023.1139521

**Published:** 2023-02-23

**Authors:** Fang Tong, Jian Lin, Zixuan Zeng, Qi Wang, Zhifang Yang, Yehui Lv

**Affiliations:** ^1^School of Basic Medical Sciences, Shanghai University of Medicine and Health Sciences, Shanghai, China; ^2^Institute of Wound Prevention and Treatment, Shanghai University of Medicine and Health Sciences, Shanghai, China; ^3^Chongming Hospital Affiliated to Shanghai University of Medicine and Health Sciences, Shanghai, China; ^4^Department of Adult Internal Medicine, Hubei Maternity and Child Health Hospital, Tongji Medical College, Huazhong University of Science and Technology, Wuhan, China

**Keywords:** SUDEP, epilepsy, bibliometric analysis, research interest, English literature, autopsy rate

## Abstract

The mechanism of sudden unexpected death in epilepsy (SUDEP) is elusive and many questions remain unanswered. Autopsy is generally unhelpful in providing evidence for the cause of death, as pathological changes may be on the molecular level. Although histopathological examination occasionally demonstrates pathology such as vascular malformation, old traumatic injury, and tumor, in most cases of SUDEP, the examination is negative. We examined the current status of SUDEP research by performing a bibliometric analysis of studies in the Web of Science Core Collection database published between 2002 and 2022. Our aim was to demonstrate areas of interest and frontiers of SUDEP research. A total of 1803 papers were included in the analysis. The number of published papers focused on SUDEP has been increasing since 2002. Main areas of interest include clinical manifestations, prevalence, treatment, and underlying mechanisms. Research teams from the United States and Europe are leading the way in SUDEP research, while Asia trails behind. Future studies regarding the mechanism and neuropathology of SUDEP are warranted.

## 1. Introduction

Public interest in sudden unexpected death in epilepsy (SUDEP) has increased in recent years (1). Autopsy findings do not distinguish SUDEP from non-SUDEP deaths in individuals with epilepsy (2). Although pathologic brain examination can occasionally identify epilepsy-related pathology such as vascular malformation, old traumatic brain injury, and brain tumor, most SUDEP cases have negative findings.

The annual incidence of SUDEP is ~1.2 in 1,000 adult patients with epilepsy (3). Previously, the incidence in children was estimated to be 0.22 per 1,000 patient-years (4, 5); however, the latest data suggests the rate is similar to that in adults (6). In a study of 1,086 SUDEP cases in the United States, males comprised 63.2% of cases and the median age at death was 39 years. Death was unwitnessed in 83.2% of cases and 77.1% occurred at home. Interestingly, ~50% of victims were found in bed and 42.4% were in the prone position (3). The incidence of epilepsy in children, adolescents and young adults has decreased over the last few decades because of improvements in medical care, sanitation, and control of infectious diseases (7). In contrast, incidence of epilepsy is increasing in the elderly because of its association with age-related diseases such as stroke and neurodegenerative disorders and recent increases in life expectancy (7). Approximately 10 million people are suffering from epilepsy in China (8), however, the detection rate of SUDEP is rarely reported.

Known mechanisms of SUDEP include persistent seizures, brain stem dysfunction, and cardiorespiratory inhibition; however, the cause of death in most cases is unexplained (9, 10). Underlying disease, fever, traumatic brain injury, drug withdrawal, infection, and metabolic insults have been reported as factors related to SUDEP. Convulsive seizure is thought to be a common risk factor. Progressive bradycardia occurs at the onset of convulsive seizure, followed by terminal apnea along with terminal asystole (11). In a mouse model of epilepsy, lesions in the ventrolateral medulla were associated with respiratory suppression in fatal seizure (12).

The diagnosis of SUDEP is challenging because no clear diagnostic criteria have been established. In the practice of legal medicine, SUDEP is a diagnosis of exclusion. Toxicology testing should be performed (2). Other potential causes of sudden death including coronary heart disease and stroke must be also eliminated. In many cases, SUDEP does not directly lead to death, however, it can give rise to accidental falls and trauma as well as drowning. The prevalence of SUDEP is underestimated (13). Previous studies have demonstrated that only ~30% of SUDEP cases are reported as SUDEP, seizure, or epilepsy (4, 14). Prevention of SUDEP-related death is imperative and has attracted worldwide concern. Primary care physicians can act to decrease epilepsy-related risks, most of which are non-neurological and preventable (1, 15).

Questions regarding the pathology, etiology, and mechanisms of SUDEP warrant further study. Here, we present a bibliometric analysis of the SUDEP literature published over the last two decades.

## 2. Materials and methods

### 2.1. Database and search strategy

We searched the Web of Science Core Collection databases, including the Science Citation Index and Social Science Citation Index, on October 1, 2022 to identify studies regarding SUDEP published from 2002 to 2022. The search strategy was (TS=sudden unexpected death in epilepsy OR TS=SUDEP) OR (TS=sudden unexpected death AND TS=epilepsy).

### 2.2. Eligibility criteria

**Figure 1:**
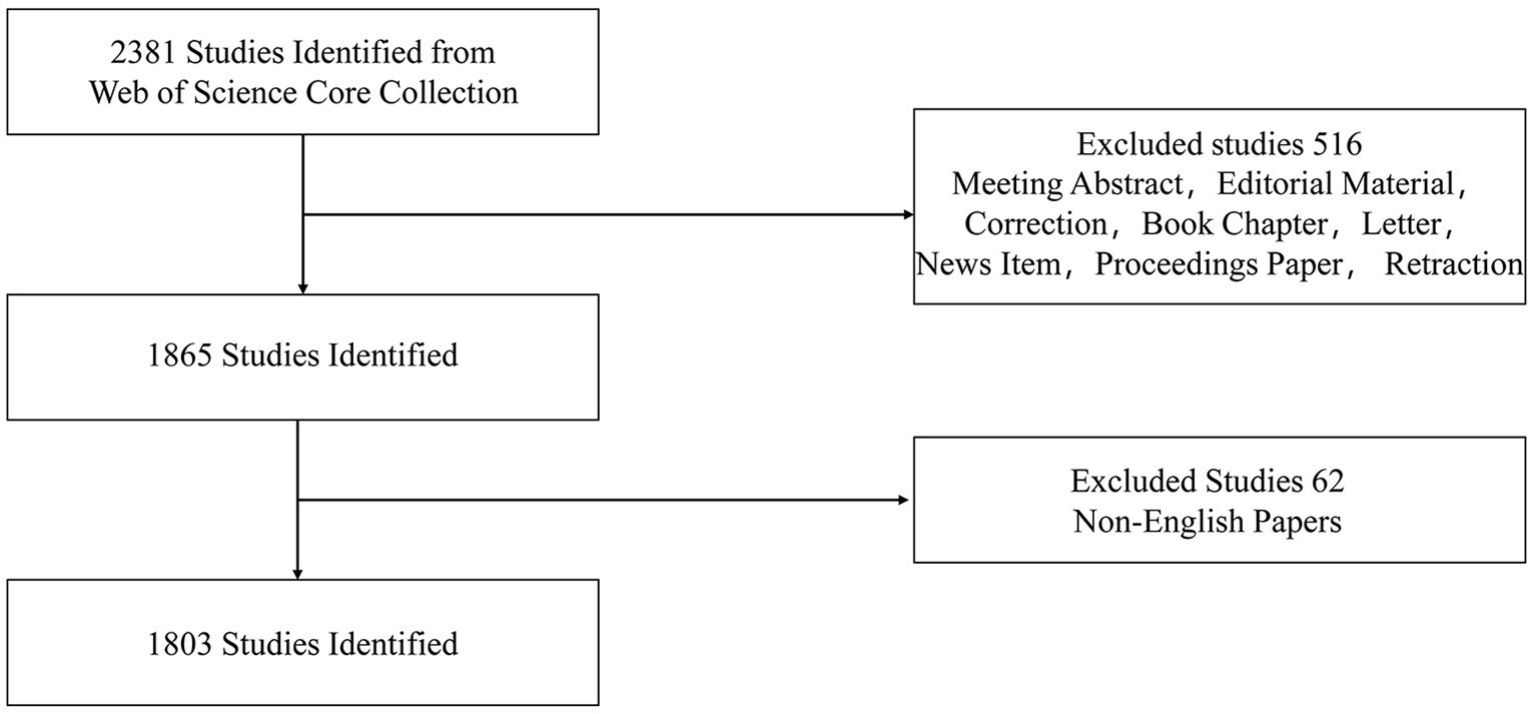
Flow chat for article selection.

Mechanism and cohort studies associated with SUDEP and published in the English language were eligible for inclusion. Publication types included original articles, reviews, meeting papers, and online publications. Meeting abstracts, editorial materials, corrections, book chapters, letters, news items, proceedings papers, and retractions were excluded. The selection process is shown in Figure 1.

### 2.3. Data analysis

CiteSpace version 5.8.2 (https://sourceforge.net/projects/citespace/postdownload) and Graphpad Prism 9 were used for data analysis after initial screening. If we did not assure the article classification of subtopics based on the metadata, the full text was read in detail for manual classification. The following indicators were chosen for analyzing research trends in SUDEP: top 10 countries and institutions for publishing SUDEP articles, top 10 journals for publishing SUDEP articles, top 10 funding sources for SUDEP studies, top 10 most cited papers and authors, and top 25 keywords. Visual maps and tables related to these indicators were created using CiteSpace.

## 3. Results

Two thousand three hundred eighty-one studies were identified and evaluated. Five hundred sixteen papers which did not meet criteria were excluded. Sixty-two non-English papers were also removed. Finally, 1,803 were included in the bibliometric analysis. Original articles (1,384, 76.761%) were most common, followed by review papers (*n* = 389, 21.575%), meeting papers (*n* = 65, 3.605%), and online publications (*n* = 5, 0.277%).

### 3.1. Growth trends of annual publication and citation number

**Figure 2:**
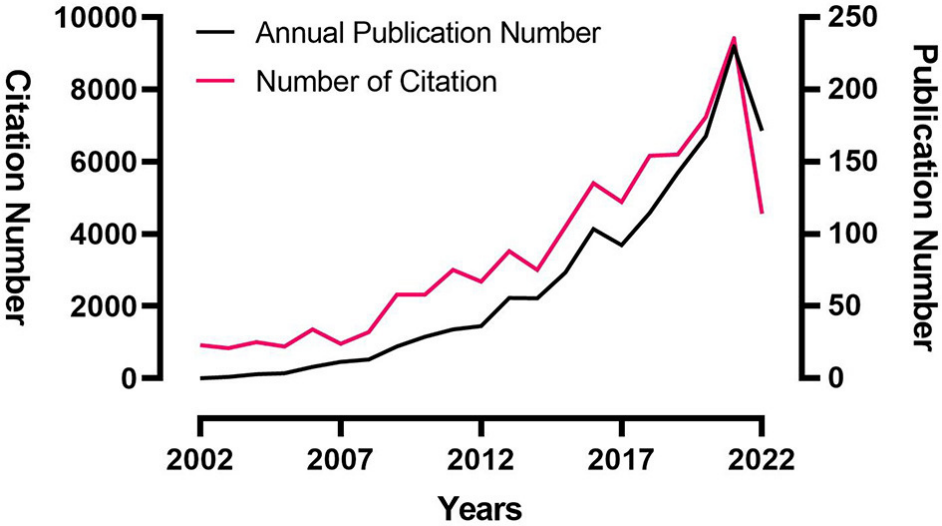
Growth trends in annual publication and citation numbers.

The growth trends of annual publication and citation numbers are shown in Table 1 and Figure 2. The number of annual publications related to SUDEP drastically increased with slight fluctuation over the last 20 years. Only 23 SUDEP papers were published in 2002, while 235 were published in 2021. One hundred fourteen articles had been published in 2022 prior to October 1. The citation number also increased from 0 in 2002 to 9178 in 2021, which paralleled the annual publication growth trend.

**Table 1:** Annual publication and citation numbers.

**Year**	**Publication number**	**Citation number**
2002	23	0
2003	21	44
2004	25	115
2005	22	141
2006	34	317
2007	24	459
2008	32	523
2009	58	880
2010	58	1,150
2011	75	1,355
2012	67	1,451
2013	88	2,226
2014	75	2,220
2015	105	2,935
2016	135	4,137
2017	122	3,693
2018	154	4,582
2019	155	5,693
2020	181	6,708
2021	235	9,178
2022	113	6,857
	h-index	100
	Self-Citation	20,639
	Sum	54,714
	Citation per paper	30.35

### 3.2. Main countries/regions and institutions

**Figure 3:**
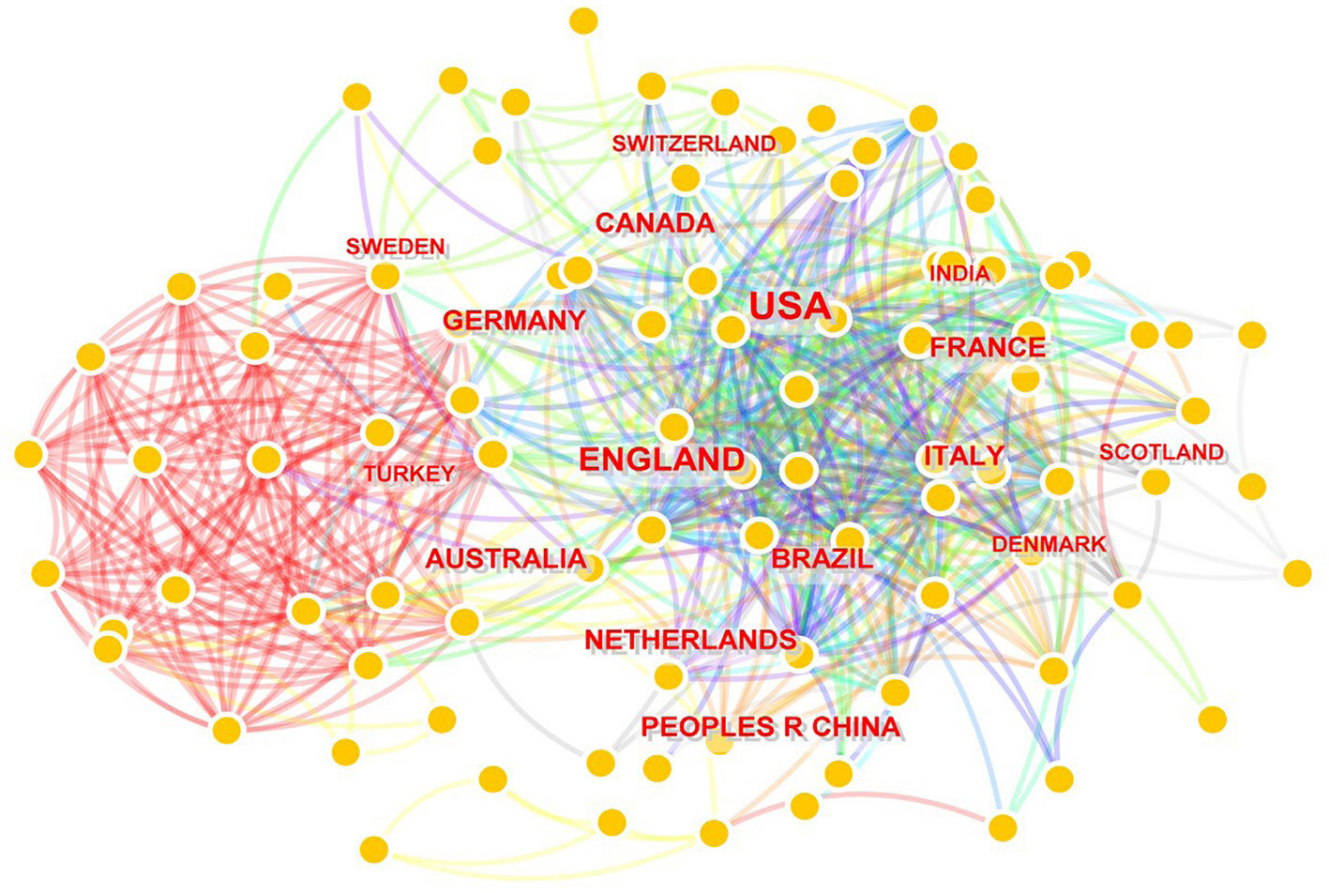
Network map of countries engaged in SUDEP research.

**Figure 4:**
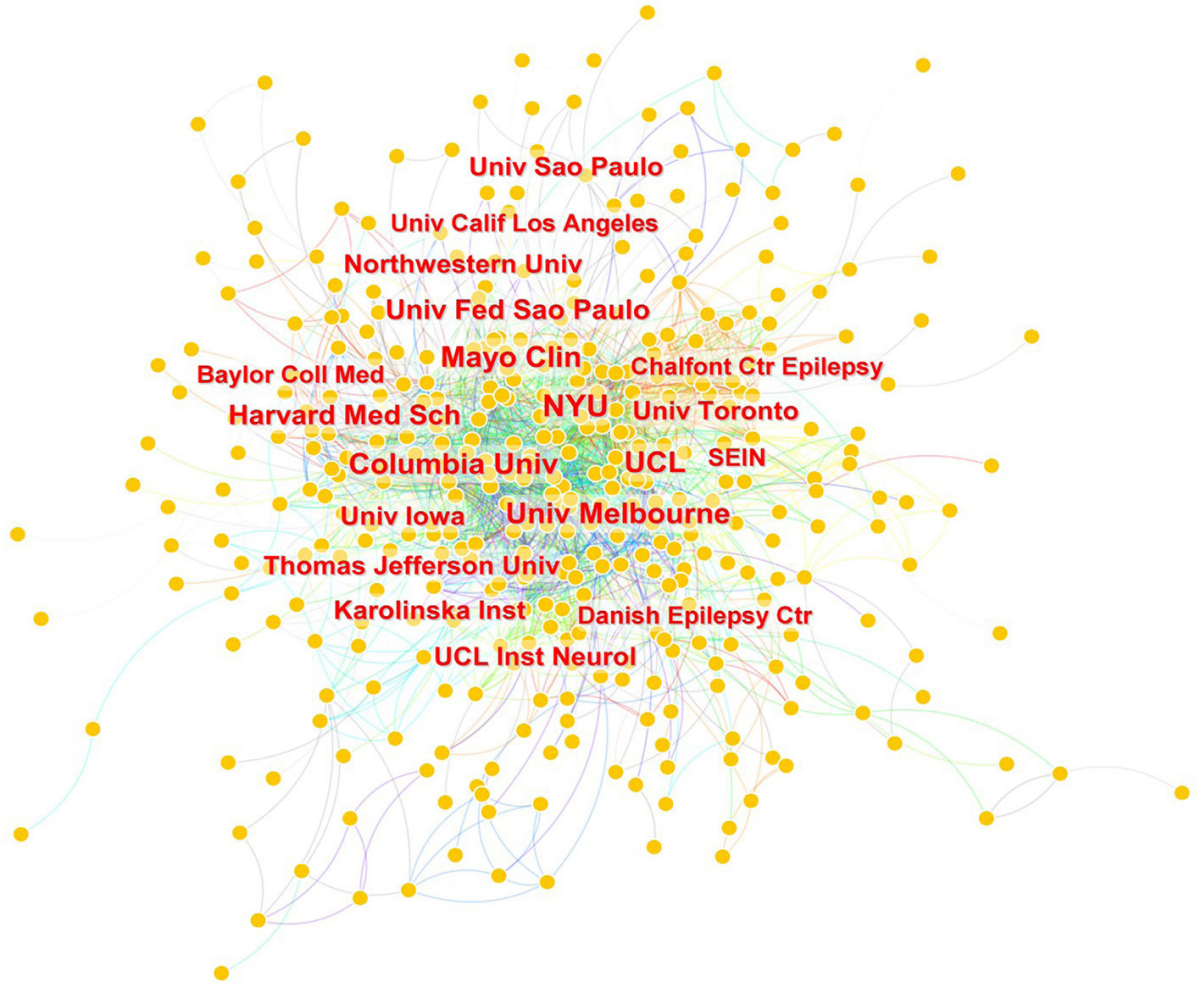
Network map of institutions engaged in SUDEP research.

A total of 95 countries/regions and 2,198 institutions contributed to 1,803 scientific articles. The countries and institutions which published the highest number of SUDEP articles from 2002 to 2022 are shown in Table 2. The United States and England were the top two countries with 790 and 275 articles, respectively. Canada, Germany, the Netherlands, Brazil, Italy, China, Australia, and France contributed 119, 109, 104, 104, 104, 103, and 89 papers, respectively during the 20 years. The top two institutions, the University of London (*n* = 179) and University College London (*n* = 156), were both in England. Most of the other institutions in the list were from the United States, including Harvard University (*n* = 100), New York University (*n* = 90), University of California System (*n* = 88), Harvard Medical School (*n* = 71), Mayo Clinic (*n* = 65), and Columbia University (*n* = 63). Network maps of countries and institutions are shown in Figures 3, 4. The top 10 countries and institutions had considerable cross-references to each other.

**Table 2:** Top 10 countries and institutions which published the highest number of SUDEP articles.

**Rank**	**1803 publications included in this study**			
	**Country**	**Count** ^a^	**Institutions**	**Count**
1	USA	790	University of London	179
2	England	275	University College London	156
3	Canada	139	Harvard University	100
4	Germany	119	New York University	90
5	Netherlands	109	University of California System	88
6	Brazil	104	Harvard Medical School	71
7	Italy	104	Universidade Federal De São Paulo Unifesp	68
8	China	104	Mayo Clinic	65
9	Australia	103	Columbia University	63
10	France	89	University of Melbourne	61

^a^The total count of countries is 2,785.

### 3.3. Primary funding agencies

Among the 1,803 analyzed papers, 340 (18.875%) were funded by the United States Department of Health and Human Services. Three hundred thirty-three (18.469%) and 224 (12.424%) were supported by the National Institutes of Health and National Institute of Neurological Disorders and Stroke of the United States. The top three funding agencies supported approximately half of the SUDEP studies. Other studies were sponsored by UCB Pharma SA (Belgium), Eisai Co., Ltd (Japan), National Institute for Health and Care Research (UK), Conselho Nacional De Desenvolvimento Cientifico E Tecnologico (Brazil), European Commission (EU), Fundacao De Amparo A Pesquisa Do Estado De São Paulo (Brazil), and GlaxoSmithKline (UK). Further details are presented in Table 3.

**Table 3:** Top 10 funding agencies for SUDEP research.

**Rank**	**Funding agency**	**Number**	**Percentage (%)**
1	United States Department of Health Human Services, USA	340	18.857
2	National Institutes of Health, USA	333	18.469
3	National Institute of Neurological Disorders Stroke, USA	224	12.424
4	UCB Pharma SA, Belgium	68	3.771
5	Eisai Co., Ltd, Japan	65	3.605
6	National Institute for Health and Care Research, UK	63	3.494
7	Conselho Nacional De Desenvolvimento Cientifico E Tecnologico, Brazil	52	2.884
8	European Commission, EU	48	2.662
9	Fundacao De Amparo A Pesquisa Do Estado De São Paulo, Brazil	46	2.551
10	GlaxoSmithKline, UK	42	2.329

### 3.4. Predominant journals

The SUDEP articles analyzed were published in 436 journals. Six journals published more than 50 papers during the 20-year period, and three published more than 100. According to Bradford's Law, 11 journals were considered core journals in the field of SUDEP (Table 4). Eight hundred and eleven papers were published in the top 11 journals, accounting for 50.5% of all. Among all the core journals, *American Journal of Forensic Medicine and Pathology* was the only one associated with forensic medicine. While the top 10 journals in the list were all specialized journals of neurology and the top three were epilepsy-specific. The impact factor of the listed journals ranged from 0.939 to 11.802.

**Table 4:** Core journals which published the highest number of SUDEP papers.

**Rank**	**Journal**	**Country**	**Count**	**Self-citation rate (%)**	**5-IF**
1	Epilepsia	USA	252	9.8	7.478
2	Epilepsy Behavior	USA	232	20.6	3.504
3	Seizure European Journal of Epilepsy	England	116	10.5	3.892
4	Epilepsy Research	Netherlands	95	5.7	3.820
5	Neurology	USA	60	5.6	11.602
6	Frontiers in Neurology	Switzerland	60	6.2	4.321
7	Epileptic Disorders	Netherlands	26	2.1	9.162
8	Arquivos De Neuro Psiquiatria	Brazil	25	7.2	1.805
9	Epilepsia Open	USA	16	7.6	2.544
10	Pediatric Neurology	USA	15	11.8	3.536
11	American Journal of Forensic Medicine and Pathology	USA	14	4.2	0.939

### 3.5. Impactful articles and authors

**Figure 5:**
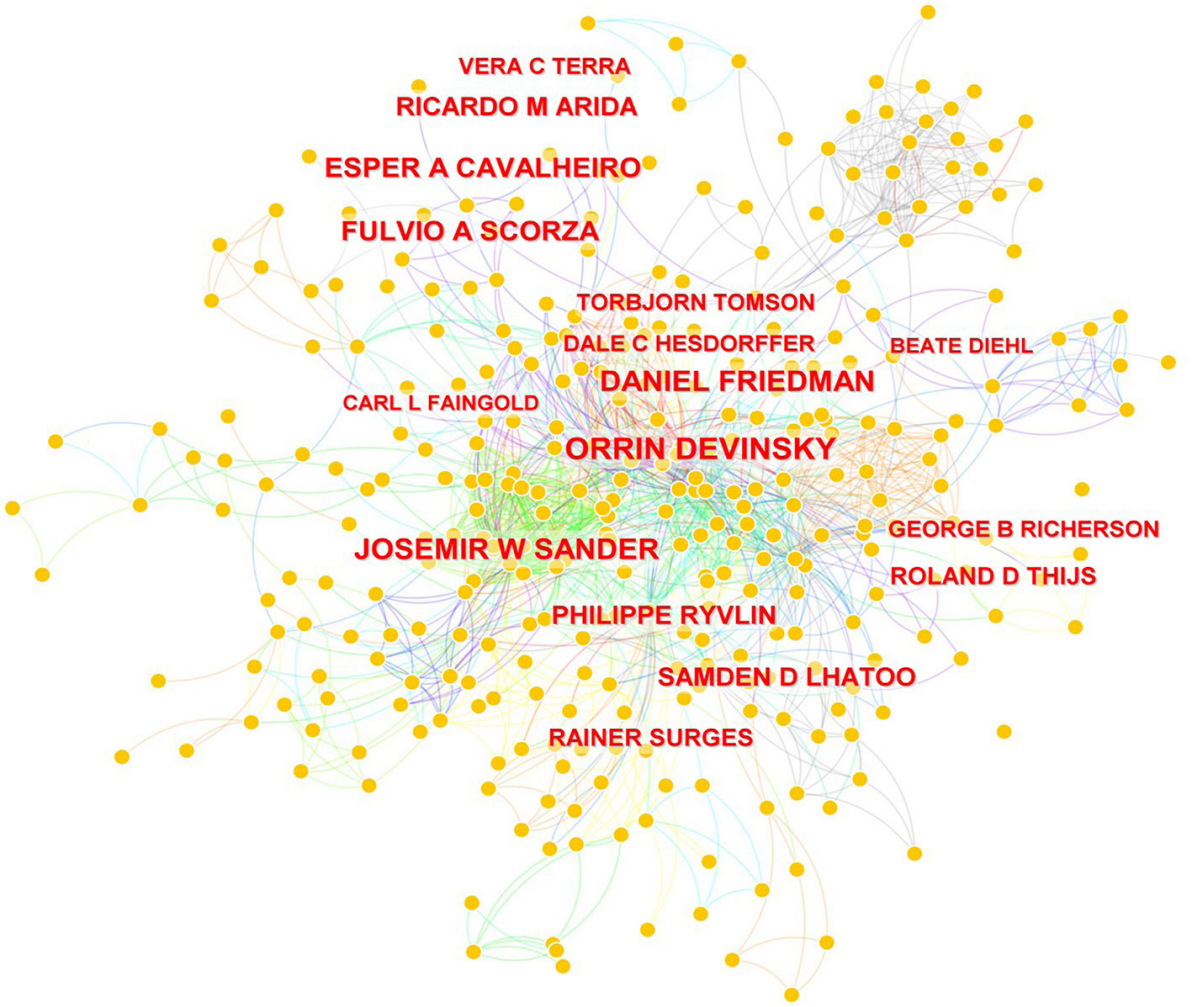
Network map of active authors contributing to SUDEP research.

Eight papers were cited more than 400 times and three more than 500. The most-cited paper was “Incidence and mechanisms of cardiorespiratory arrest in epilepsy monitoring units (MORTEMUS): a retrospective study,” which was published in Lancet Neurology in 2013 and cited 611 times. The journals which published the top 10 most-cited articles included Lancet Neurology, Epilepsia, Lancet, Current Opinion in Neurology, Journal of the American College of Cardiology, and International Epilepsy & Behavior. The most-cited papers are listed in Table 5. The impact of the 742 authors who published SUDEP research is visualized in Figure 5, which demonstrates the co-occurrence among authors. The impact of an author is positively associated with the name size exhibited in the figure. Orrin Devinsky, Josmir W Sander, Daniel Friedman, Fulvio A Scozar, and Esper A Cavalheiro, were the top impactful authors.

**Table 5:** Top 10 impactful SUDEP papers.

**Rank**	**Most cited articles**					
	**First author**	**Title**	**Journal**	**Year**	**Number of citations**	**Citations per year**
1	Ryvlin, P	Incidence and mechanisms of cardiorespiratory arrests in epilepsy monitoring units (MORTEMUS): a retrospective study (11)	LANCET NEUROLOGY	2013	611	61.1
2	Thurman, DJ	Standards for epidemiologic studies and surveillance of epilepsy (16)	EPILEPSIA	2011	598	49.8
3	Duncan, JS	Adult epilepsy (17)	LANCET	2006	557	32.8
4	Sander, JW	The epidemiology of epilepsy revisited (18)	CURRENT OPINION IN NEUROLOGY	2003	554	27.7
5	Devinsky, O	Cannabidiol in patients with treatment-resistant epilepsy: an open-label interventional trial (19)	LANCET NEUROLOGY	2016	503	71.9
6	Moshe, SL;	Epilepsy: new advances (20)	LANCET	2015	486	60.8
7	Tomson, T	Sudden unexpected death in epilepsy: current knowledge and future directions (21)	LANCET NEUROLOGY	2008	467	33.6
8	Thijs, RD	Epilepsy in adults (22)	LANCET	2019	444	111.0
9	Stecker, EC	Population-based analysis of sudden cardiac death with and without left ventricular systolic dysfunction - Two-year findings from the Oregon sudden unexpected death study (23)	JOURNAL OF THE AMERICAN COLLEGE OF CARDIOLOGY	2006	349	20.5
10	Laxer, KD	The consequences of refractory epilepsy and its treatment (24)	INTERNATIONAL EPILEPSY & BEHAVIOR	2014	241	34.4

### 3.6. Top keywords and analysis

**Figure 6:**
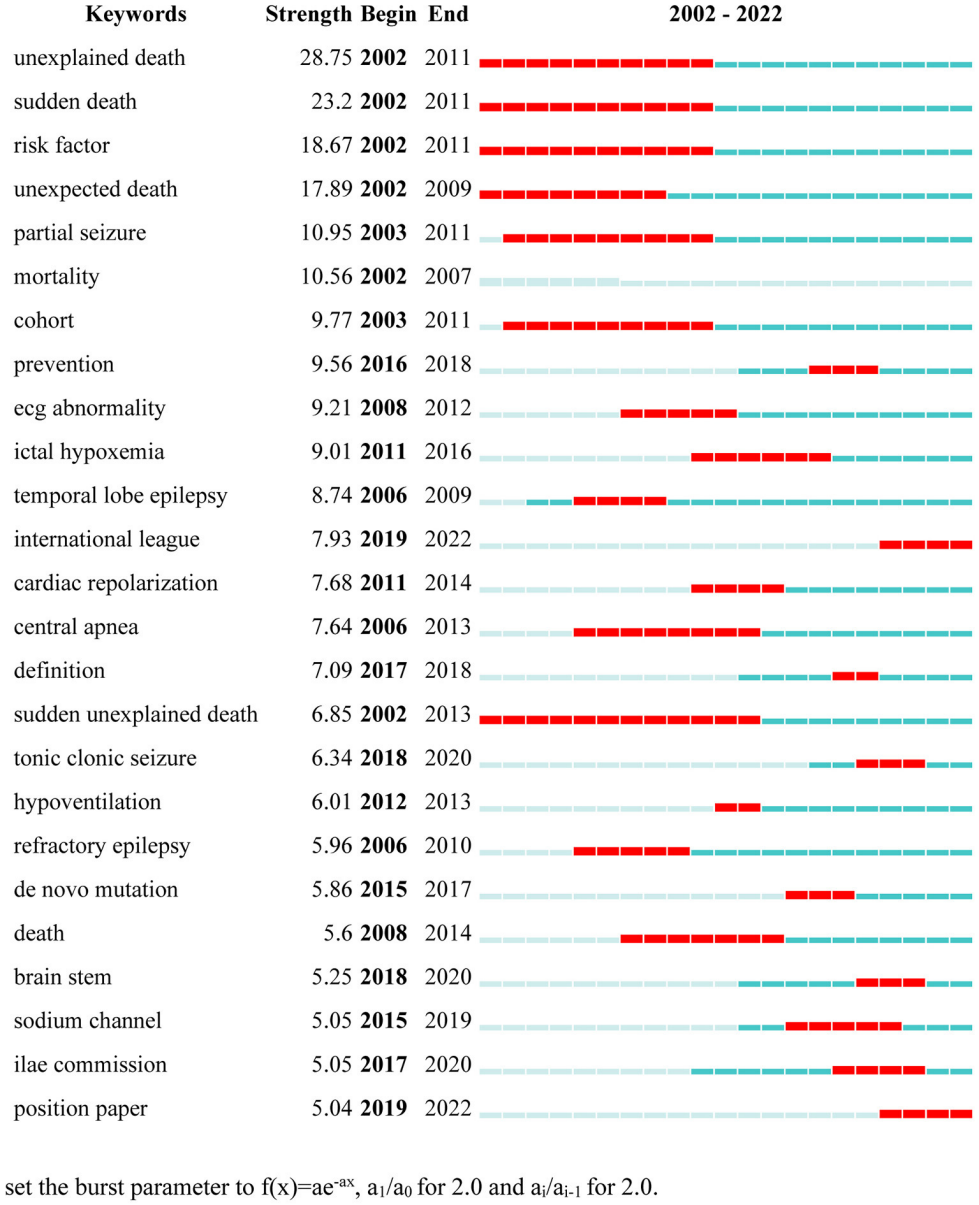
Top 25 keywords with the strongest citation burst.

Keywords were extracted from the SUDEP articles and 25 burst words were exported after CiteSpace analysis. Keywords with the strongest citation burst are listed in Figure 6. Burst words significantly changed over the 20-year period. “Unexplained death” and “sudden death” were the top two strongest keywords in the list, emerging from 2002 to 2010 and 2002 to 2011, respectively, with burst intensities of 28.75 and 23.2. The third-ranked keyword was “risk factor” with a strength of 18.67.

**Figure 7:**
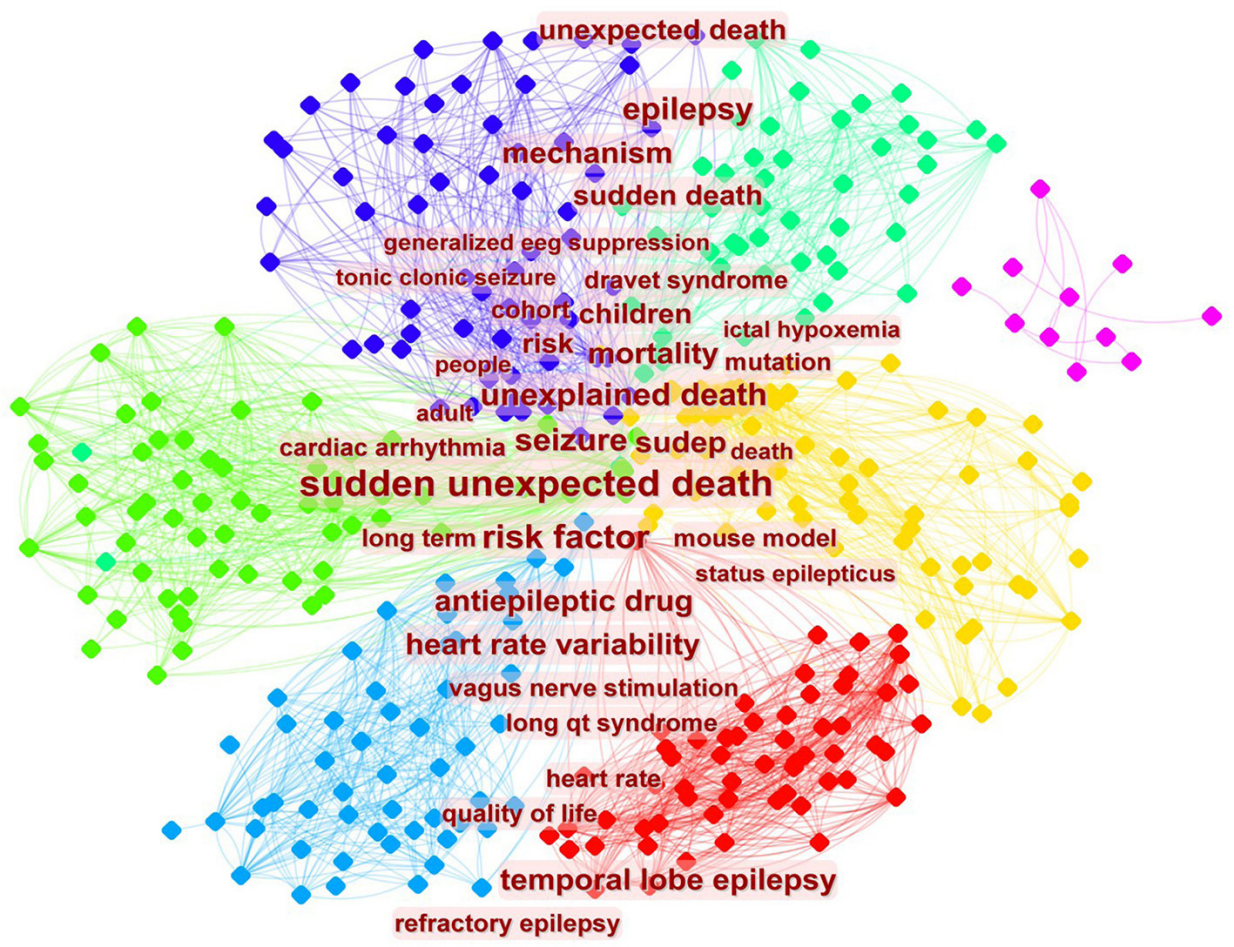
Co-occurrence map of keywords.

Clustering analysis on the co-occurrence of keywords is shown in Figure 7. The figure demonstrates many important issues of co-occurrence such as sudden unexpected death, seizure, unexplained death, children, risk factor, heart rate variability, temporal lobe epilepsy, and mechanism.

## 4. Discussion

The results from this bibliometric analysis demonstrate that SUDEP attracted growing research attention from 2002 to 2022. The number of SUDEP articles published in 2021 was 10 times higher than that published in 2002. The burst of papers is partly related to increasing support from official agencies. National Institutes of Health funding for SUDEP increased by 10,000-fold between 2009 and 2016 (3), and the number has increased by 2.5-fold from 2016 to 2022 (25). United States funding agencies supported the most SUDEP research, followed by the United Kingdom, Japan, European Union, and Brazil. Chinese funding agencies did not make it into the top 10, which is unparallel to their greater support for other fields of research (26).

From 2002 to 2022, the United States and European countries were the predominant drivers in SUDEP research. However, developing countries including China and Brazil also participate. Both countries contributed 104 articles over the 20-year period examined and rank in the top 10. Although institutions from the United States published the highest number of articles in the period, the top two institutions are located in the United Kingdom and three institutions from the top 10 are in London. Institutions from the United States account for six positions in the top 10. An institution from Brazil, Universida de Federal de São Paulo, published 68 papers in the 20-year period and is ranked seventh; this was the only developing country institution in the top 10. Surprisingly, institutions from the European Union were absent from the top 10.

Our results also demonstrate that the most important journals publishing SUDEP research are Epilepsia and Epilepsy Behavior. Epilepsia published the highest number of articles, accounting for 14%. The top 10 journals combined published 49.7% of all SUDEP papers. Most journals publishing SUDEP articles focus on neurology and some specialize in epilepsy or seizure. Diagnosis of SUDEP is extremely difficult in the practice of forensic medicine and should be paid great attention. *American Journal of Forensic Medicine and Pathology* is one of the core journals of SUDEP according to the Bradford's Law, publishing 14 papers regarding SUDEP in the two decades. We hope that more practitioners in forensic medicine can take part in the research of SUDEP.

The most impactful article was “Incidence and mechanisms of cardiorespiratory arrests in epilepsy monitoring units (MORTEMUS): a retrospective study,” written by Ryvlin et.al. They retrospectively collected and analyzed data from all cardiorespiratory arrests associated with SUDEP in numerous epilepsy monitoring units in Europe, Israel, Australia, and New Zealand (11). They proposed improving supervision at night to prevent immediate cardiorespiratory dysfunction induced by unpredictable seizures (11). The second most-cited paper was a guideline, ‘Standards for epidemiologic studies and surveillance of epilepsy' published in Epilepsia. In this article (16), Thurman et.al. discussed the following issues: (1) conceptual and operational definitions of epilepsy, (2) data resources and recommended data elements, and (3) methods and analyses appropriate for epidemiologic studies or epilepsy surveillance. The paper is instructional for designing studies with different purposes and provides variant methods for data retrieval based on the particular needs of different study teams.

Keyword frequency can reflect study interests within a particular research field. According to our results, the hotspots in SUDEP research predominantly focus on mortality, epidemiology, and clinical manifestations. The results demonstrated in the list of burst keywords are in accordance with the hotspots figure. Most research did not pay attention to the etiology of SUDEP. Issues such as disease mechanism and neuropathology were not sufficiently studied during the period, which hinders a deeper understanding of the disease. Although postmortem examinations are likely to be unhelpful in SUDEP cases, occasionally they can provide interesting findings. In one case report (27), a capillary telangiectasia was observed in the hippocampus in a patient who died of unexplained seizures, implying that hippocampal pathology could induce fatal epilepsy. Some study teams start to study SUDEP at the molecular level in the near 10 years. Indeed, keywords of *de novo* mutation and sodium channel emerged in 2015, which suggests that research on SUDEP is reaching the deep-water zone. Overall, the etiology of SUDEP includes a series of different factors and cannot be explained by a single theory. Despite the low detection rate, we still recommend performing systematic pathological examination in all suspected cases of SUDEP. Furthermore, brain tissue, cerebrospinal fluid, and serum should be stored in a special SUDEP tissue bank to enable further studies at the molecular level.

We also propose that the Chinese government increase SUDEP funding and increase the autopsy rate in SUDEP cases. A better understanding of SUDEP can prolong life expectancy and enhance quality of life. The low autopsy rate is hindering China from making progress in SUDEP as well as other fields of research. Developed countries pay greater attention to autopsies for all deaths: in 2019, the autopsy rate in the Commonwealth of Nations and the World Health Organization European Region was 43.4 and 25.9%, respectively (28). In the United States, the autopsy rate was 50% in the 1940s but drastically declined to 8% in 2018 (29). Only 1.6% of all deaths in Japan were autopsied in 2014, the lowest among developed countries (30). Unfortunately, the percentage in China is even lower—from 1990 to 2011, the autopsy rate in large teaching hospitals in China was <1% and some hospitals performed no autopsies (31). Therefore, we can speculate that the overall autopsy rate in China is extremely low. If China increased the national autopsy rate to 10%, they would be able to make considerable contributions to SUDEP research.

## 5. Conclusion

The number of published papers focused on SUDEP has been increasing since 2002. Current study interests involve clinical manifestations, prevalence, treatment, and underlying mechanisms. Research teams from the United States and Europe are leading the way in SUDEP research, while Asia trails behind. Systematic pathological examination could increase the understanding of SUDEP. Tissue banks of SUDEP could be established to enable further studies at the molecular level. Studies which focus on mechanisms and neuropathology are warranted to improve our understanding of this disease.

## Data availability statement

The raw data supporting the conclusions of this article will be made available by the authors, without undue reservation.

## Author contributions

FT, YL, and ZY designed the framework of the review and drafted the manuscript. FT, ZZ, JL, ZY, YL, and QW provided supervision and contributed to manuscript writing and editing. All authors have read and approved the latest version of the manuscript.
